# Perceived Social Support and Its Effects on Changes in the Affective and Eudaimonic Well-Being of Chilean University Students

**DOI:** 10.3389/fpsyg.2020.590513

**Published:** 2020-12-11

**Authors:** Rubia Cobo-Rendón, Yaranay López-Angulo, María Victoria Pérez-Villalobos, Alejandro Díaz-Mujica

**Affiliations:** ^1^Laboratorio de Investigación e Innovación educativa Dirección de Docencia, Universidad de Concepción, Concepción, Chile; ^2^Facultad de Ciencias Sociales y Comunicaciones, Universidad Santo Tomas, Concepción, Chile; ^3^Facultad de Ciencias Sociales, Departamento de Psicología, Universidad de Concepción, Concepción, Chile

**Keywords:** perceived social support, eudaimonic well-being, university students, longitudinal study, affective well-being

## Abstract

The beginning of university life can be a stressful event for students. The close social relationships that they can experience can have positive effects on their well-being. The objective of this paper is to estimate the effect of perceived social support on the changes of the hedonic and eudaimonic well-being of Chilean university students during the transition from the first to the second academic year. Overall, 205 students participated (63.90% men and 36.09% women) with an average age of 19.14 years (*SD* = 1.73), evaluated during their first academic year (2017) and the succeeding one (2018). For the evaluation of perceived social support, the Spanish version of the Perceived Social Support Questionnaire “MSPSS” was used, and PERMA-profiler was used to measure hedonic and eudaimonic well-being. Changes through the time of hedonic well-being and social support and the correlations between the variables were analyzed. Changes in the perception of social support were analyzed according to four categories of hedonic well-being. The prediction of social support for eudaimonic well-being was evaluated. Results indicated that the perception of students’ social support did not change over time. Statistically significant differences were found in hedonic well-being scores in the two measurements, being significantly higher in the first measurement than in the second one. More than 50% of the participants presented a positive balance of affections. The perception of social support is associated with the two types of well-being. Students who had a high balance of affections had a greater perception of general social support than the groups of positive evolution of affections and a low balance of affections. In the case of the friends and family support dimensions, the perception in the high-balance group of affections concerning the low-scale group is greater. Improving the perception of social support increases the eudaimonic well-being of university students. The perception of support that students had during the beginning of their university life benefits their general well-being, which contributes to their mental health.

## Introduction

There is currently an interest in the study of mental health issues in Higher Education. Research in different contexts describes the importance of mental health in university students (Wörfel et al., 2016; Leung, 2017), with well-being being an important aspect in students’ academic success (Langford et al., 2014).

The concept of well-being can be evaluated from the hedonic and eudaimonic approach. Hedonic well-being is defined as the presence of positive moods, the absence of negative moods (affective components), and life satisfaction (cognitive component) related to life assessment (Diener et al., 1999). Affective balance, part of this type of well-being, is defined as the ability to balance the emotions associated with life experiences. It is an affective element resulting from the estimation of the differences between the presence of positive and negative emotions experienced by each person over time (Diener, 2000; Diener et al., 2010, 2015).

Affective well-being can be defined as frequent experiences of pleasant moods, such as joy, and infrequent experiences of negative affect or unpleasant moods, such as anger and fear (Diener et al., 2010). This type of well-being is measured by the frequency and intensity of positive and negative emotions. Leaving aside the idea of a single construct of bipolar affect (pleasant affect versus unpleasant affect), the affective component considers evaluations of both, positive and negative affective states. That is, the presence of a positive affect is not the same as the absence of a negative affect (Diener et al., 2017; Weismayer, 2020). An important aspect to consider is that positive life events are closely related to pleasant emotions, whereas negative life events are accompanied by unpleasant emotions (Diener, 2000). Due to this, affective well-being has been associated with better health and longevity (Diener and Chan, 2011). Regarding temporality, the presence of various moods in cognitive evaluation is the result of the experiences presented in people’s lives (Diener et al., 1999). Several studies indicate a greater presence of positive than negative affectivity in university students and the general population (Contreras et al., 2010; Diener et al., 2010; Zubieta et al., 2012; Barrantes-Brais and Ureña-Bonilla, 2015). Correlations among positive affectivity and academic performance, hope, curiosity, enthusiasm, and perseverance were found in the literature review regarding Higher Educational Contexts (Oriol, 2016; Zhang and Chen, 2018).

Eudaimonic well-being is defined as the cognitive assessment of the development of skills and virtues necessary for optimal psychological development (Keyes et al., 2002; Ryff, 2014). It is related to the search for optimal personal development or human flourishing (Waterman, 2008), with the meaning of life, personal growth, autonomy, positive relationships with others, self-esteem, and cognitive flexibility (Ryff and Singer, 1996; Ryff, 2014; Malkoç and Mutlu, 2019).

One of the most widely accepted theoretical approaches was the one presented by Carol Ryff, who defines psychological well-being from six dimensions: self-acceptance: positive self-evaluations, positive relationships: satisfactory and authentic interpersonal relationships, autonomy: sense of self-determination and independence, mastery of the environment: ability to effectively manage one’s life and the surrounding world, personal growth: sense of continuous personal development and life purpose, which is considered as the sense of self-direction, and persistence in fulfilling vitally important goals (Ryff, 1989). In longitudinal terms, the well-being profiles that are persistently high or low are considered as predictors of chronic diseases and functional deterioration concerning health subjective terms (Ryff et al., 2015). Additionally, there is consensus in considering the relevance of close, deep, personal sense social relationships in well-being and other mental health indexes (Feeney and Collins, 2015; Poots and Cassidy, 2020).

People with close social relationships tend to report higher levels of well-being and flourishing (Diener and Seligman, 2002; Myers, 2015; Diener et al., 2018). Perceived social support affects the way people perceive themselves and the world around them. A meta-analysis indicates that not having a network of meaningful relationships in life is more predictive of mortality than other lifestyle behaviors, such as smoking or physical activity (Holt-Lunstad and Smith, 2012).

Social support is the perception of being cared for by others and having a reliable network to turn to when needed, in everyday situations or specific moments of crisis (Taylor, 2011). It can be perceived from three sources: family, friends, and significant others (Zimet et al., 1988). Social support is also referred to as the frequency of support actions that are provided by others (Santini et al., 2015); which is why, it can be understood as the subjective feeling of being supported (Santini et al., 2015). Additionally, the type of support can be (1) emotional, (2) instrumental, (3) evaluative, and (4) informative (Sarason et al., 1990).

Overall, perceived social support is a significant predictor of life satisfaction and negative affect (Siedlecki et al., 2014; Kostak et al., 2019; Shensa et al., 2020). Specifically, emotional support has important benefits in mental health, so many studies focus on the relationship between depression and perceived social support (Kleiman and Riskind 2013; Santini et al., 2015; Kostak et al., 2019; Shensa et al., 2020). Perceived social support and social bonds are positively related to mental and physical health (Cohen and Janicki-Deverts, 2009; Umberson and Karas Montez, 2010). Research points to a positive association between perceived social support and psychological well-being, which allows it to be seen as a valuable protective mechanism that can improve psychological well-being by maintaining positive emotional feelings and mitigating stress (Chu et al., 2010; Thoits, 2011; Liu et al., 2014). The different facets of hedonic well-being (life satisfaction, positive and negative affects) can be predicted by different aspects of perceived social support (Siedlecki et al., 2014). Also, perceived social support is seen as mediating the relationship between stress and well-being (Poots and Cassidy, 2020).

Research on predictors of academic success points to perceived social support as one of the facilitating factors of adaptation to university life and protective of challenging situations imposed by the university (Friedlander et al., 2007; Bahar, 2010; Rahat and İlhan, 2016). Since the theory of self-determination, the need to belong to a group is relevant for healthy psychological development and human flourishing (Ryan and Deci, 2000; Tomás and Gutiérrez, 2019); however, little has been studied about the effects of perceived social support over time.

Due to affective well-being implying the presence of positive and negative affects, with no exclusion of both previous components (Diener et al., 2017; Weismayer, 2020), it is important to investigate how the perception of social support that was acquired during the first year of the university experience can affect the students’ well-being. Although research has linked perceived social support to well-being measures, some researchers have found negative or no consequences of perceived social support on well-being (Lepore et al., 2008; Lakey et al., 2010). Differences in outcomes can be derived from the ways in which perceived social support and well-being are conceptualized and operationalized (Siedlecki et al., 2014), and research on these issues has also focused on cross-sectional measures. For this reason, it is necessary to evaluate how the student’s perception of social support affects his/her well-being, considering the process that he/she undergoes during university entry and the empirical evidence that first-year students have the lowest levels of well-being (Brandy et al., 2015; van Der Zanden et al., 2018). Thus, this paper aims at estimating the effect of perceived social support on changes in affective and eudaimonic well-being in university students.

To respond to this objective, the following hypotheses are raised. First, the perception of social support and affective well-being in participating university students varies over time (H1). Second, there is a positive relationship between the perception of social support with affective and eudaimonic well-being (H2). Third, perceptions of social support predict changes over time in affective well-being and eudaimonic well-being in university students (H3).

## Materials and Methods

The research design used corresponds to a comparative-prospective study, and at a temporal level, it is a longitudinal panel research (Ato et al., 2013).

### Participants

Overall, 131 (63.90%) were men, and 74 (36.09%) were women. The average age was *M* = 19.14 years, *SD* = 1,730. Follow-up was achieved in only 26.3%, and there were cases of some careers where it was not possible to perform follow-up applications due to problems of access to student groups due to academic activity stoppages, impacting on the percentage of students analyzed in Q2.

The distribution of participants who were followed up by degree courses is as follows: 56.09% (*n* = 115) belonged to Engineering and Basic Sciences, 18.04% (*n* = 37) to Architecture, 14.14% (*n* = 29) to Social Sciences and Humanities, and 11.70% (*n* = 24) to Pedagogy or Education. The selection was through a non-probability sample for convenience, considering the first-year students of the participating careers.

### Instruments

#### Perceived Social Support

The Perceived Social Support Questionnaire “MSPSS” (Zimet et al., 1988), specifically the Spanish version by Ortíz and Baeza (2011), was used for the evaluation of perceived social support. The purpose of this questionnaire is to assess people’s perception of social support from relevant sources. It is designed as a self-report and is composed of 12 items distributed in three dimensions: family (four items), e.g., “My family gives me the help and emotional support I need”; friends (four items), e.g., “I can talk about my problems with my friends”; and other significant ones (four items), e.g., “When I have difficulties I have someone to support me.” This version uses a Likert response scale and ranges from 1 = strongly disagree to 7 = strongly agree. The higher the score, the higher the perception of social support. In this study, confirmatory factor analysis corroborated the distribution of three related factors [X2(51) = 123,269, *p* < 0.05; root mean square error of approximation (RMSEA) = 0.084; Confirmatory Fit Index (CFI) = 0.935; Tucker-Lewis Index (TLI) = 0.916]. The reliability was from *ω* = 0.89 for the family dimension, from *ω* = 0.91 for the friend’s dimension, and from *ω* = 0.89 for the other significant dimensions in the first measurement.

#### Emotional and Eudaimonic Well-Being

The PERMA-Profiler (Butler and Kern, 2016) was used for the well-being evaluation. This instrument consists of 23 items with a Likert response format where 1 = lowest score and 7 = highest score. It is made up of five main dimensions (positive emotions, commitment, positive relationships, purpose, and achievement) and three contrast dimensions (negative emotions, health perception, and one item for loneliness). For this study, the dimensions were reorganized to evaluate the elements of affective well-being (positive and negative affect) and eudaism separately. The coincidence of the dimensions proposed by the PERMA-Profiler, to the concepts to the theory of psychological well-being, such as the meaning of life, autonomy, and positive relations with others, allows the identification and measurement of this variable (Ryff and Singer, 1996; Ryff, 2014).

The eudaimonic well-being was measured with 12 items that correspond to: the dimension of positive relationships with others (three items), evaluates to what extent the person receives and gives support to others, their satisfaction, and the feeling of being appreciated, e.g., how often do you feel loved; the meaning or purpose of life (three items), involves the feelings of leading a life with intention, meaning, and value, e.g., in general, how often do you feel you are following a meaningful direction in your life; the competence or achievement dimension (three items), assesses how often the person advances in their goals, takes responsibility, and feels able to complete their daily responsibilities, e.g., how often are you able to handle your responsibilities?; and the commitment dimension (three items), assesses the presence of vigor, absorption, and dedication, e.g., how often do you feel absorbed in what you are doing. The average of the dimensions forms an indicator of psychological well-being with a range of responses from 1 to 7 points. Other studies report adequate psychometric properties in university students and Latin American samples (Butler and Kern, 2016; Pastrana and Salazar-Piñeros, 2016; Lima-Castro et al., 2017). To confirm these dimensions, a confirmatory factorial analysis was performed; an adequate model fit was obtained [X2(48) = 91,748, *p* < 0.05; RMSEA = 0.067; CFI = 0.947; TLI = 0.927]. The reliability of the scale ranged from *ω* = 0.89 in the first measurement.

Emotional well-being was measured with six items from the same questionnaire, of which three measure positive emotions of satisfaction, joy, and optimism, e.g., in general, how often do you feel happy, and the remaining three measure negative emotions, such as anxiety, sadness, and anger, e.g., in general, how often do you feel angry? Each emotion has a range of responses between 1 and 7 points. The subtraction between positive and negative emotions is used as an indicator of the scale of affect. A confirmatory factor analysis was performed to confirm these dimensions, obtaining an adequate model fit [X2(8) = 16,677, *p* < 0.05; RMSEA = 0.073; CFI = 0.973; TLI = 0.949]. The reliability for positive emotions was *ω* = 0.84 and negative *ω* = 0.67 in the first measurement.

#### Procedure

The present research was endorsed by the Ethics Committee of the participating university, and the ethical criteria for research with human beings were corroborated. During the applications, participants were informed of the characteristics of the study, the right to voluntary participation, and the handling of data privacy. For the collection of data, contact was made with the authorities of the faculties (Deans, Department Directors, and Heads of Studies), who indicated the courses where the applications would be made. Participants were selected through a non-probabilistic sampling for convenience. The questionnaires were massively applied in the classrooms during March 2017 (Q1). The follow-up was carried out during April and June 2018 (Q2) in the same careers that were applied in Q1. The difference was that the classrooms where the applications were made corresponded to the courses of second-year students, since 1 year had passed.

For the follow-up (Q2), the data identifying the participants who answered the instruments in the classrooms during Q2 were compared with the database obtained in Q1. Three identification criteria were used (first and last names, ID card, and career). Based on these criteria, follow-up cases were digitally identified, and also, a manual check of the data set was performed.

#### Analysis Plan

The MPLUS software was used to carry out the confirmatory factor analyses of the scales. The SPSS Windows version 21 software was used for data analysis descriptive (means, standard deviations, frequencies, percentages) to describe the characteristics of the sample and the main variables of the study. SPSS was also used to analyze changes over time in emotional well-being and the perception of social support (H1), with Student’s *t*-test of related samples. Pearson correlations were made to estimate the relationships of perception of social support, affective well-being, and eudaimony (H2). To respond to H3, participants were initially classified into four categories of affective well-being according to the balance of affects scores obtained in the two measurements, with possible combinations being made for high and low balance of affects cases. Subsequently, factor-type variance analyses (ANOVAs) were performed to evaluate the prediction of perceived social support in the types of affective and eudaimonic well-being (H3).

## Results

The general objective of this study was to estimate the effect of perceived social support on changes in the emotional and eudaimonic well-being of university students. Table 1 describes the scores obtained for the dimensions of perceived social support and types of well-being.

**Table 1:** Scores on social support, affective, and eudaimonic well-being.

Dimensions	T1		T2		*t* (gl)	Sig.
	Mean	*SD*	Mean	*SD*		
Social support: friends	5.63	1.19	5.52	1.21	1.451 (198)	0.148
Social support: family	5.63	1.30	5.49	1.33	1.843 (201)	0.067
Social support: others	5.69	1.18	5.67	1.10	0.506 (199)	0.613
Affective well-being	4.06	5.54	2.60	5.84	4.191 (195)	0.001
Eudaimonic well-being	5.00	0.89	4.91	0.92	1.245 (195)	0.215

When performing the means comparison analysis to confirm whether the perception of social support and the subjective well-being vary over time (H1), it was found that the social support dimensions did not present statistically significant changes over time. Concerning the affective well-being, statistically significant differences were found in the scores of T1 and T2 being significantly higher in the scores presented at the beginning of the academic year (*M* = 4.06, *SD* = 5.54) than in the second year (*M* = 2.60, *SD* = 5.84).

To confirm the hypothesis (H2), which describes positive relations of the perception of social support with the types of well-being, Pearson’s correlation estimates are described in Table 2. The results indicate a higher level of association of T1 perceived social support with the eudaimonic and affective well-being in T1 than with the scores obtained in T2.

**Table 2:** Pearson’s correlation of the dimensions of perception of social support with affective and eudaimonic well-being in university students.

	1		2		3		4		5		6		7		8		9	
1. S.S. friends T1	–																	
2. S.S. family T1	0.42	***	–															
3. S.S. others T1	0.73	***	0.53	***	–													
4. S.S. friends T2	0.62	***	0.37	***	0.46	***	–											
5. S.S. family T2	0.24	***	0.62	***	0.29	***	0.46	***	–									
6. S.S. others T2	0.53	***	0.43	***	0.61	***	0.75	***	0.52	***	–							
7. W.B. affective T1	0.37	***	0.38	***	0.37	***	0.25	***	0.20	**	0.23	**	–					
8. W.B. affective T2	0.13		0.25	***	0.20	**	0.26	***	0.31	***	0.26	***	0.63	***	–			
9. W.B. eudaimonic T1	0.48	***	0.47	***	0.53	***	0.37	***	0.29	***	0.39	***	0.63	***	0.40	***	–	
10. W.B. eudaimonic T2	0.23	***	0.29	***	0.32	***	0.44	***	0.32	***	0.45	***	0.42	***	0.64	***	0.58	***

S.S., perception of social support; W.B., well-being.

^*^The correlation is significant at level 0.05 (bilateral).

** The correlation is significant at level 0.01 (bilateral) .

*** The correlation is significant at level 0.001 (bilateral).

When analyzing the correlations, at a cross-sectional level, the results confirm positive and significant correlations between the dimensions of perceived social support with affective well-being and eudaimonic well-being (T1). The magnitude of the correlations of the perception of social support was higher with the eudaimonic well-being in the T1 and T2 measurements. A greater relationship was found between the dimensions of the perception of social support (T1) and the eudaimonic well-being (T2) than with the affective well-being (T2); meanwhile, there is no statistically significant correlation in the dimension of the perception of social support of friends (T1) with the affective well-being (T2). At a general level, there is more congruence between the dimensions of perceived social support and eudaimonia well-being in all its measurements.

### Changes in the Perception of General Social Support According to the Evolution of Emotional and Eudaimonia Well-Being

To assess the prediction of social support in the change of students’ well-being (H3), four categories of well-being change were established (see Table 3). In this case, the group of students with “low well-being stable” refers to the group of students who presented low scores (less than 3pts) in the two measures. The group “negative change in well-being” in the first measurement presented high scores (higher than 3pts) and in the second one low scores. The category called “positive evolution of well-being” includes the group of students who presented low scores in the first measurement and in the second one high scores; finally, the category “stable high well-being” includes the students who presented high scores during the two measurements. In the case of affective well-being, the results presented in Table 3 indicate that more than 50% of the participants present a positive affective balance. In the case of eudaimonic well-being, 38% present high scores in the second measurement.

**Table 3:** Change in the balance of well-being during the beginning of the university career.

Change in scores	Frequency	Percentage
**Affective well-being**		
Low stable well-being	33	16.0
Negative evolution of well-being	34	16.5
Positive evolution of well-being	15	7.3
High stable well-being	114	55.3
Total	196^*^	95.1
**Eudaimonic well-being**		
Low stable well-being	11	5.3
Negative evolution of well-being	5	2.4
Positive evolution of well-being	6	2.9
High stable well-being	74	35.9
Total	96^**^	46.6

* 9 students with incomplete answers.

** 110 students with incomplete answers.

**Figure 1:**
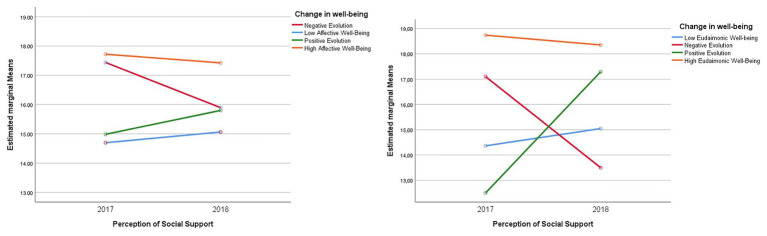
Differences in the perception of general social support by categories of evolution of affective and eudaimonic well-being.

ANOVA Factorials were carried out to respond to this hypothesis. When analyzing the differences in the perception of social support by categories of the evolution of affective well-being, statistically significant differences are presented in the scores of the perception of social support in the analyzed affectivity groups *F*(3,187) = 4.264, *p* < 0.01, *ηp*^2^ = 0.064 (see Figure 1). Additionally, statistically significant inter-subject effects are presented *F*(3,187) = 10.187, *p* < 0.001, *ηp*^2^ = 0.140.

Multiple comparisons obtained from Bonferroni’s *post-hoc* tests indicate that students in the high affective well-being category maintain similar scores of high perception of general social support during the two measurements (*M* = 17.72, *SD* = 2.88 and *M* = 17.42, *SD* = 2.89, respectively). Similarly, it is observed that the averages in the categories of students of positive evolution of affective well-being (*M* = 14.98, *SD* = 3.47 and *M* = 15.80, *SD* = 3.47) and low stable affective well-being (*M* = 14.69; *SD* = 3.14 and *M* = 15.06; *SD* = 3.00) had at the beginning of the first year a perception of social support different from the one they presented in the second academic year).

In the case of the perception of social support by categories of the evolution of eudaimonic well-being, statistically significant differences are presented *F*(3,187) = 4.264, *p* < 0.01, *ηp*^2^ = 0.064 (see Figure 1). Additionally, there are statistically significant inter-subject effects *F*(1,89) = 20.590, *p* < 0.001, *ηp*^2^ = 0.410.

Multiple comparisons obtained from Bonferroni’s *post-hoc* tests indicate that students in the high well-being category of eudaimonics have higher scores for general social support during the two measurements (*M* = 18.73, *SD* = 1.94 and *M* = 18.5, *SD* = 2.08, respectively). Unlike the rest of the categories that presented changes in the two measurements, for example, positive evolution of well-being (*M* = 12.500, *SD* = 3.77 and *M* = 17.29, *SD* = 1.998), a group of students who presented an increase in the perception of social support as their well-being also increased. In the case of the group of students in the category “negative evolution of well-being” (*M* = 14,100, *SD* = 1.48 and *M* = 13,500, *SD* = 1.944), their perception of support decreased. In the case of the category of stable low well-being, students in this category did not show substantial changes over time (*M* = 14,363, *SD* = 3.16 and *M* = 15,045, *SD* = 2,431). Differences between the groups were found, as students from the high stable well-being group presented a higher perception of social support than the rest of the students belonging to the other categories (*p* < 0.01); the description of the changes found is reflected in Figure 1.

### Changes in the Perception of Social Support From Friends According to the Evolution of Emotional and Eudaimonic Well-Being

**Figure 2:**
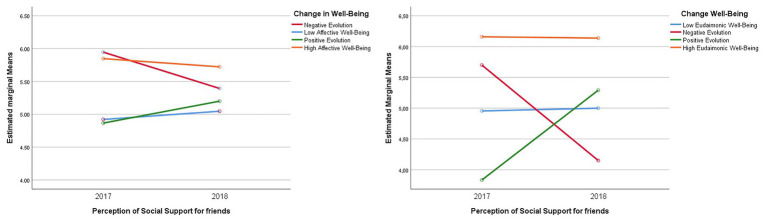
Differences in the perception of social support from friends by categories of evolution of affective and eudaimonic well-being.

The results found to analyze the differences in the perception of social support from friends according to the categories of affective well-being indicate statistically significant differences in the scores of perceived social support in the analyzed affectivity groups *F*(3,188) = 3.459 (*p* < 0.01), *ηp*^2^ = 0.052 (see Figure 2). Statistically significant inter-subject effects are presented *F*(3,188) = 6.436, *p* < 0.001, *ηp*^2^ = 0.093. According to the results of Bonferroni’s *post-hoc* test, statistically significant differences (*p* < 0.01) were found only between the groups of students in the high affective stable well-being category (*M* = 5.84, *SD* = 1.03 and *M* = 5.72, *SD* = 1.14), with the low affective stable well-being group (*M* = 4.92, *SD* = 1.39 and *M* = 5.04, *SD* = 1.35), respectively. In this case, the last group of students shows an increase in their scores over time (see Figure 2).

In the case of the perception of social support from friends by categories of evolution of eudaimonic well-being, statistically significant differences are presented *F*(1,90) = 11.006, *p* < 0.01, *ηp*^2^ = 0.268 (see Figure 2). Additionally, there are statistically significant inter-subject effects *F*(1,90) = 214.267, *p* < 0.001, *ηp*^2^ = 0.322.

Multiple comparisons obtained from Bonferroni’s *post-hoc* tests indicate that students in the high well-being category of eudaimonics have higher scores for general social support during the two measurements (*M* = 6.159, *SD* = 0.874 and *M* = 6.138, *SD* = 0.708, respectively). Unlike the rest of the categories that presented changes in the two measurements, e.g., positive evolution of well-being (*M* = 12,500, *SD* = 3.77 and *M* = 17.29, *SD* = 1.998), as in the case of the stable low well-being category, students did not present substantial changes over time (*M* = 5,000, *SD* = 1.17 and *M* = 4.954, *SD* = 1.223). In the case of the group of students in the “negative well-being development” category (*M* = 5,700, *SD* = 480 and *M* = 4,150, *SD* = 1,024), their perception of support decreased. Differences between the groups were found, as students from the high well-being group with stable debt presented a higher perception of social support than the rest of the students belonging to the other categories (*p* < 0.01); the description of the changes found is reflected in Figure 2.

### Changes in the Perception of Social Support From the Family According to the Evolution of Emotional Well-Being

**Figure 3:**
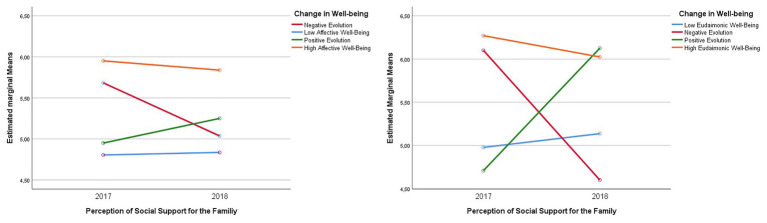
Differences in the perception of social support from the family by categories of evolution of affective and eudaimonic well-being.

In the case of differences in the perception of social support from family members according to the categories of affective well-being, statistically significant differences were identified in the scores of perceived social support of this dimension in the affectivity groups analyzed *F*(3,190) = 3,242, *p* < 0.05, *ηp*^2^ = 0.049 (see Figure 3). Additionally, statistically significant inter-subject effects are presented *F*(3,190) = 9.788, *p* < 0.001, *ηp*^2^ = 0.134. Statistically significant differences (*p* < 0.01) were only found between the groups of students in the category of high stable affective well-being (*M* = 5.95, *SD* = 1.18 and *M* = 5.83, *SD* = 1.11) and those of low stable affective well-being (*M* = 4.80, *SD* = 1.18 and *M* = 4.83, *SD* = 5.95), respectively. In this case, this last group of students shows an increase in their scores over time (see Figure 3).

In the case of the perception of social support from the family by categories of evolution of eudaimonic well-being, statistically significant differences are presented *F*(1,91) = 11,372, *p* < 0.01, *ηp*^2^ = 0.273 (see Figure 3). Additionally, there are statistically significant inter-subject effects *F*(1,91) = 7.557, *p* < 0.001, *ηp*^2^ = 0.199.

Multiple comparisons obtained from Bonferroni’s *post-hoc* tests indicate that students from the high stable eudaimonic well-being category present higher scores of general social support during the two measurements (*M* = 6.270, *SD* = 0.783 and *M* = 6.1024, *SD* = 0.966, respectively). In the low stable well-being category, students did not present substantial changes over time (*M* = 4.977, *SD* = 1.221 and *M* = 5.136, *SD* = 1.062). The rest of the categories that presented changes in the two measurements, e.g., positive evolution of well-being (*M* = 4,708, *SD* = 1,111 and *M* = 6,125, *SD* = 0.786) and in the group of students in the category “negative evolution of well-being” (*M* = 6,100, *SD* = 0.894 and *M* = 4,600, *SD* = 1,126), whose perception of support decreased. Differences between the groups were found, as students from the high well-being group with stable debt presented a higher perception of social support than the rest of the students belonging to the other categories (*p* < 0.01); the description of the changes found is reflected in Figure 3.

With regard to the identification of changes in the perception of social support from other significant people according to the evolution of affective and eudaimonic well-being, no statistically significant differences were found in the groups evaluated.

## Discussion

The aim of this work was to estimate the effect of perceived social support on changes in the emotional and eudaimonic well-being of university students. The results found allowed to confirm that the perception of social support positively predicts the types of well-being of Chilean university students. Next, the findings obtained in relation to the established hypotheses are analyzed, and the limitations presented are commented on and future lines of research that could be projected from the results of this study.

### Changes Over Time in the Perception of Social Support and Subjective Well-Being

In this study, only temporary changes were identified in the emotional well-being scores, partially confirming the H1 hypothesis (the perception of social support and emotional well-being in the participating university students varied over time). This result confirms findings of Joshanloo (2018a,b), who refers to which elements of emotional well-being can change due to situations presented in the context, generating changes in people’s moods. This result is congruent with what is established in the theory about affective well-being, where the result of the evaluation that a person makes by means of cognitive and affective processes is associated to his life experiences (Diener et al., 1999).

Positive emotions are considered to be part of the affective component of well-being, which can vary over the course of a day (Pavot and Diener, 2011). When students present these types of emotions, they are better able to handle the new academic demands present at the university during the first years (Ouweneel et al., 2011; Oriol-Granado et al., 2017). The results found in the present study indicate higher affective balance scores at the beginning of the university career (during the beginning of the first academic year), allowing people to expand their repertoire of thoughts and behavior, building their lasting social and personal resources (Fredrickson, 2013). The presence of high affective balance scores at the beginning of the career could be related to the search for immediate well-being, rather than a long-term search (Joshanloo, 2018b).

The decline in measurement scores in Q2 is similar to that reported in other studies. Herke et al. (2019) found a decline in affective well-being in students. Importantly, changes in students’ affect balance may depend on their perception of the context (Lent et al., 2005). The decrease in students’ affective well-being can be explained by the adaptation processes they undergo at the beginning of their university careers. Nightingale et al. (2013) report that when students present low levels of positive emotions, they manifest difficulties in the process of adaptation to university. In that sense, during the first years of the university experience, students tend to adjust their beliefs, knowledge, and perceptions with respect to the real academic context, and it is possible that students experience stressful experiences that can impact on their affectivity (Nicolson, 1990; Coffey et al., 2016; Kyndt et al., 2017).

### Relationship Between the Perception of Social Support and Emotional and Eudaimonic Well-Being

As for the existence of a positive relationship between the perception of social support with the affective and eudaimonic well-being (H2), the results allowed to confirm the hypothesis. Positive relations are presented between the dimensions of the perception of social support with the types of well-being. In general, the perception of social support is considered as a protective factor for the well-being of university students (Rosa-Rodríguez et al., 2015). Perceived social support allows to re-evaluate stressful situations contributing to adaptation processes in different contexts (Pillado and Almagiá, 2019).

When assessing relationships specifically, the results show that during Q1 and Q2, the perception of social support from friends, family, and significant others is more related to eudaimonic well-being than to affective well-being. Therefore, family support is more linked to elements associated with personal growth and the optimal development of students. This result coincides with what is reported in the literature, and studies show a close relationship between perceived social support and eudaimonic well-being (Almagia, 2012; Pillado and Almagiá, 2019). Family support and peer support influence the psychological well-being of university students, which, in turn, has a strong positive correlation with improved academic performance (Torales et al., 2018).

The presence of positive relationships between perceived social support and emotional well-being is also consistent with other research establishing that: when students perceive greater social support from family and friends, they report greater satisfaction with life (Marrero and Carballeira, 2010; Kong et al., 2012; Kong and You, 2013; King et al., 2020). There is a consensus that close, deep and meaningful social relationships have a positive effect on subjective well-being and other mental health indices (Dávila Figueras et al., 2011; Feeney and Collins, 2015; Poots and Cassidy, 2020). Additionally, the distinction found in the levels of relationship of perceived social support with types of well-being coincides with another study that establishes that the prediction of the perception of social support can vary depending on the well-being indicator taken into account. Although this variable has an important weight on life satisfaction and psychological adjustment, it explains to a lesser extent the presence of positive or negative affectivity and has little impact on satisfaction (Marrero and Carballeira, 2010).

### Predicting the Perception of Social Support in Affective and Eudaimonic Well-Being

The results found confirm the H3 hypothesis that the perception of social support predicted changes over time in affective well-being and changes in eudaimonic well-being in university students. It has been proposed that the perception of social support is vital for well-being (Diener and Oishi, 2005). In other studies, perceived support was a predictor of both increased life satisfaction and decreased negative affect (Siedlecki et al., 2014). Similarly, emotional well-being was affected by the amount of social support, and low levels of social support received were more associated with depressive symptoms (Ye et al., 2019).

As reported in the literature, student groups with high stable and positive emotional well-being had a higher perception of overall social support than groups with lower levels of emotional well-being. This coincides with the results presented by Siedlecki et al. These authors establish that people who have satisfactory relationships report feeling happy more often and report being more satisfied with their lives than those who do not have satisfactory relationships. This is possible because people in satisfying relationships can get support when they need it, whereas those in unsatisfying relationships cannot easily get it when they need it (Siedlecki et al., 2014). It is clear that perceived social support affects the way people perceive themselves and the world around them; having a network of meaningful relationships in life has positive effects on people’s physical and mental health (Holt-Lunstad and Smith, 2012).

A statistically significant effect was found on emotional well-being and changes in the perception of social support from friends and family. There were differences between the groups with high emotional well-being and the group with low emotional well-being. Our results are similar to those found in other studies that establish that perceived social support from friends is positively related to well-being. Such findings reinforce the idea of the adaptive value of peer support in the Higher Education setting (Figueira et al., 2017).

In the case of the perception of social support coming from the family, the findings are similar to those described in other studies that explain that the family represents integral support in different aspects of the life of university students, who even after entering university and feeling adult, continue to consider the family as a valuable source to support them in their needs (Monroy and Ramírez, 2016). Students live a process where the development of a progressive autonomy is encouraged (Barrera-Herrera and Vinet, 2017); however, when they are overtaken by different demands typical of their stage, they may turn to their parents for support (Barrera-Herrera et al., 2019). Studies report that the support received from the family benefits the self-perceived academic performance of university students (López-Angulo et al., 2020). In this sense, the perceived social support exercised by family and friends is an essential element for the success of students (Renk and Smith, 2007; Torales et al., 2018).

Concerning perceptions of social support as predictors of eudaimonic well-being in university students, the results of this research allowed us to conclude that well-being increases from a greater perception of the social support that students have. The relationship between the perception of social support and eudaimonic well-being is explained by the importance for students of developing social networks to help them cope with the demands of the university stage (Rosa-Rodríguez et al., 2015). It is possible to consider perceived social support as a valuable protection mechanism, which can improve eudaimonic well-being (Chu et al., 2010; Thoits, 2011; Liu et al., 2014), benefiting personal growth, autonomy, and cognitive flexibility, which improves the response to the demands of university life (Waterman, 2008; Ryff, 2014, 2016; Malkoç and Mutlu, 2019).

A limitation of this research is the non-inclusion of sociodemographic, mental health (e.g., stress, depression, coping strategies), and contextual variables (e.g., semester assessment period), which could have influenced students’ well-being scores. The analysis could also be enriched by incorporating other educational indicators, such as objective academic performance or levels of adaptation to university (Bailey and Phillips, 2016; Biasi et al., 2018). Despite these limitations, the main strength of this study is the use of a longitudinal research design for the evaluation of these variables.

The results obtained have practical implications since social support among peers works as a positive element for the development of students’ emotional well-being. It is suggested to reinforce in academic institutions the development of social interventions and programs (e.g., initiation ceremonies and mentoring projects with the active participation of older students, activities involving the family), in order to raise the well-being of young university students (Figueira et al., 2017). Future student development programs should focus on improving or enhancing the adaptive capacities of regulating emotions and promoting reciprocal social exchanges (Ye et al., 2019).

## Data Availability Statement

The raw data supporting the conclusions of this article will be made available by the authors, without undue reservation.

## Ethics Statement

The studies involving human participants were reviewed and approved by the Ethics Committee of the participating university. The patients/participants provided their written informed consent to participate in this study.

## Author Contributions

RC-R contributed to the literature, abstract, and full-text review, as well as the data extraction, the data analysis, and the writing of the manuscript. YL-A contributed to the design of the study, abstract, and full-text review, as well as the design of the statistical analysis and the writing of the manuscript. MP-V contributed to the design of the study, the interpretation of the results, and the writing of the manuscript. AD-M contributed to the interpretation of the results and the writing of the manuscript. All authors contributed to the article and approved the submitted version.

### Conflict of Interest

The authors declare that the research was conducted in the absence of any commercial or financial relationships that could be construed as a potential conflict of interest.
